# Effect of erythromycin and rifampicin on monoethylglycinexylidide test

**DOI:** 10.4103/0253-7613.41044

**Published:** 2008

**Authors:** Satish Balkrishna Bhise, Remeth Jacky Dias

**Affiliations:** Govt. College of Pharmacy, Vidyanagar, Karad, (MS) - 415 124, India; 1 Satara College of Pharmacy, Plot No 1539, New Add MIDC, Degaon, Satara, Maharashatra - 415 004, India

**Keywords:** CYP3A4, lidocaine, monoethylglycinexylidide, metabolism

## Abstract

**Background:**

The dynamic liver function test based on the hepatic conversion of lidocaine to monoethylglycinexylidide (MEGX) provides a direct measure of the actual functional state of the liver. Cytochrome P450 (CYP) 3A4 has been proposed as the main CYP isoform responsible for MEGX formation. The concomitant use of either CYP3A4 inducer rifampicin or CYP3A4 inhibitor erythromycin may influence the results of MEGX test. Hence, the objective of this study was to evaluate the effect of a CYP3A4 inhibitor erythromycin and inducer rifampicin on the MEGX test.

**Materials and Methods:**

The study included 20 healthy male volunteers whose routine laboratory tests were normal. As per study protocol, MEGX test was carried out in all the participants after an overnight fast. All the participants were given 1 mg/kg lidocaine dose intravenously and MEGX concentration at 30 and 60 min after IV dose was measured using HPLC. These MEGX values served as control values. Ten subjects received 600 mg/day erythromycin orally for six days while remaining ten participants received 600 mg/day rifampicin orally for six days. On the sixth day, MEGX test was carried out two hours after the last dose.

**Result:**

Rifampicin increased the mean plasma concentration of MEGX_30_ from 93.94 ± 26.31 to 98.54 ± 24.94 μg/ml (*P* = 0.085) and MEGX_60_ from 134.34 ± 35.42 to 136.36 ± 33.14 μg/ml (*P* = 0.051). Erythromycin lowered the serum concentration of MEGX_30_ from 101.37 ± 39.39 to 96.67 ± 36.09 μg/ml (*P* = 0.128) and MEGX_60_ from 142.52 ± 42.65 to 138.98 ± 40.23 μg/ml (*P* = 0.159).

**Conclusion:**

It can be concluded from this study that the MEGX test is not affected by concomitant administration of CYP3A4 modifiers rifampicin and erythromycin.

The dynamic liver function test based on the hepatic conversion of lidocaine to monoethylglycinexylidide (MEGX) provides a direct measure of the actual functional state of the liver. Hence, MEGX test has found widespread application for real time assessment of hepatic function in cirrhosis, transplantation and critical care medicine.[1] The principal metabolic pathway of lidocaine in human beings is oxidative de-ethylation to MEGX, which is further de-ethylated to glycinexylidide (GX). The latter is hydrolyzed to xylidine and then oxidized to 4-hydroxy-xylidine, the main metabolic product found in urine.[2]

Cytochrome P450 (CYP) 3A4 has been proposed as the main CYP isoform responsible for MEGX formation.[3] Rifampicin has been a potent inducer of the CYP3A4 isoenzyme while erythromycin has been shown to produce quasi-irreversible inhibition of CYP3A4 *in vitro*.[4] So the concomitant use of either CYP3A4 inducer rifampicin or CYP3A4 inhibitor erythromycin may influence the results of MEGX test. The previous study by Isohanni *et al*,[5] showed that erythromycin and itraconazole increases the concentration of MEGX. This may affect MEGX test results. Hence, the objective of the present study was to assess the possible interference of rifampicin and erythromycin with MEGX test.

## Materials and Methods

### Subjects

The study included 20 healthy male volunteers who underwent a thorough clinical examination, including medical history, physical examination and standard laboratory tests, viz. alanine aminotransferase (ALT), aspartate aminotransferase (AST), alkaline phosphatase (AP), total bilirubin (TB), albumin (ALB), and prothrombin time (PT). All participants were non-smokers, non-alcoholics and were not taking any drugs. A written informed consent was obtained from all participants. The study protocol was approved by the institutional ethics committee.

### Study design and procedures

The experimental protocol after an overnight fast was started at 8.00 am. On day one, all participants were given 1 mg/kg lidocaine intravenously, slowly over 2 min. After lidocaine administration, all subjects remained supine for two hours. They were asked to report any subjective adverse effects and their vital signs were closely monitored. Venous blood samples were stored at −20%C until analysis. MEGX concentrations were taken as control values.

Ten subjects were given 600 mg/day erythromycin orally and the remaining ten received 600 mg/day rifampicin orally for six days. On the sixth day, MEGX test was performed two hours after the last dose. A meal low in protein and fat contents was provided during the study so as to prevent food- induced changes in liver blood flow.

MEGX concentration was determined by high performance liquid chromatography method with ultraviolet detection as described by Chen *et al*.[6] The calibration graph was linear over the range 4-250 ng/ml with intra- and inter-assay relative standard deviations of 8% and 9% respectively. The limit of detection (LOD) for the method was 3 ng/ml while limit of quantification (LOQ) was 4 ng/ml. The preliminary experiments showed that rifampicin and erythromycin did not interfere with the assay method used for analysis.

### Statistical analysis

Statistical analyses were performed with the Graph Pad Prism 4 package. A power analysis, based on the coefficients of variation previously obtained for the 30-60 min MEGX concentrations,[7] indicated that ten subjects should be sufficient to detect differences of 20% with a significance level of 0.05 and power of 80%, in both, rifampicin and erythromycin studies. MEGX concentration values from 30-60 min were tested for normal distribution using the method of Kolmogorov and Smirnov. As normal distribution of the data would not be rejected, the comparisons were made using paired t-test and later confirmed by Wilcoxon signed rank test. A *P* value of < 0.05 was considered statistically significant. The results are given as mean ± standard deviation (SD).

## Results

Twenty healthy male volunteers with mean age of 33.1±9.5 years were studied whose laboratory results for ALB, TB, AST, ALT, AP and PT were within the normal range. No significant changes in blood pressure or pulse rate were observed after lidocaine injection. Three subjects experienced mild and transient adverse effects like numbness, lightheadedness, vertigo and drowsiness, which lasted for 2-3 min following lidocaine injection.

The mean MEGX concentrations before and after P450 induction with rifampicin treatment are shown in Table 1 and those with erythromycin treatment are shown in Table 2. The mean MEGX_30_ and mean MEGX_60_ concentrations were increased by 4.6 ng/ml and 2.01 ng/ml respectively, after P450 induction with rifampicin. However, these increments were not significant (MEGX_30_: *P*value = 0.085, MEGX_60_: *P*value = 0.051). Likewise, mean MEGX_30_ and mean MEGX_60_ concentrations were decreased by 4.8 ng/ml and 3.5 ng/ml respectively, after P450 inhibition with erythromycin. Here also, the decrease in MEGX concentration was found to be non-significant (MEGX_30_: *P*value = 0.128 and MEGX_60_:*P*value = 0.159), as given in table 2.

**Table 1:** Monoethylglycinexylidide concentrations before and after P-450 induction by rifampicin

*Monoethylglycinexylidide concentrations in healthy volunteers (n = 10)*					

*Lidocaine i.v. 1 mg/kg*	*Before rifampicin (Mean ± SD)*	*After rifampicin (600 mg 6 days p.o.) (Mean ± SD)*	*Mean difference (95%CI)*	*P value*	*Remarks*
MEGX_30 min_	93.94 ± 26.3	98.54 ± 24.94	−4.6(−9.98 to 0.78)	0.085	N.S.
MEGX_60 min_	134.35 ± 35.42	136.37 ± 33.14	−2.01(−4.92 to 0.89)	0.051	N.S.

SD: standard deviation, N.S.: not significant, CI: confidence interval

**Table 2:** Monoethylglycinexylidide concentration before and after P-450 inhibition by erythromycin

*Monoethylglycinexylidide concentration in healthy volunteers (n = 10)*					

*Lidocaine i.v. 1 mg/kg*	*Before erythromycin (Mean ± SD)*	*After erythromycin (600 mg 6 days p.o.) (Mean ± SD)*	*Mean difference (95%CI)*	*P value*	*Remarks*
MEGX_30 min_	101.54 ± 39.3	96.68 ± 36.0	4.8(−1.69 to 11.41)	0.128	N.S.
MEGX_60 min_	142.53 ± 42.6	138.99 ± 40.2	3.5 (−1.68 to 8.76)	0.159	N.S.

SD: standard deviation, N.S.: not significant, CI: condidence interval

## Discussion

This study was performed to assess the effect of CYP3A4 modifiers, erythromycin and rifampicin in MEGX test. Healthy male volunteers were chosen because the hepatic effects of P-450 induction or inhibition are more pronounced in healthy individuals than in patients with impaired liver function.[8] This study has shown that concomitant administration of rifampicin increases MEGX_30_ and MEGX_60_ values but the effect is not statistically significant. Likewise, concomitant administration of erythromycin decreases MEGX concentration but not significantly. The modest effect of these modifiers on lidocaine metabolism may be due to the following reasons:

1. Lidocaine has high hepatic extraction ratio of 62-81% therefore its systemic clearance depends more on liver blood flow than metabolic capacity and consequently may not be very sensitive to the action of metabolic modifiers.[9]
2. The lidocaine - deethylation capacity of the healthy human liver may not be saturated after lidocaine doses which are used in the MEGX test. Therefore, induction of the CYP3A4 mediated lidocaine - deethylase activity may not be directly related to changes in MEGX plasma concentrations after lidocaine i.v.[10]
3. Other CYP isoforms may contribute to lidocaine biotransformation. The recent studies have shown that CYP1A2 catalyses the 3- hydroxylation of lidocaine biotransformation and is also involved in its de-ethylation.[11,12] The study by Orlando *et al*[13] have further concluded that MEGX formation is mainly catalyzed by CYP1A2 rather than CYP3A4. The authors have used CYP1A2 inhibitors, fluvoxamine in the study to show lidocaine- fluvoxamine interaction.

It can be concluded that CYP3A4 inducer, rifampicin and inhibitor, erythromycin do not influence MEGX test. This study correlates well with other studies[14–16] concluding that the metabolism of lidocaine is based on both CYP1A2 and CYP3A4 isoforms rather than that of CYP3A4 alone.
